# miRNAs Participate in the Regulation of Oxidative Stress-Related Gene Expression in Endometrioid Endometrial Cancer

**DOI:** 10.3390/ijms232415817

**Published:** 2022-12-13

**Authors:** Paweł Mieszczański, Szmon Januszyk, Nikola Zmarzły, Piotr Ossowski, Konrad Dziobek, Dorota Sagan, Dariusz Boroń, Marcin Opławski, Beniamin Oskar Grabarek

**Affiliations:** 1 Hospital of Ministry of Interior and Administration, 40-052 Katowice, Poland; 2 ICZ Healthcare Hospital in Zywiec, 34-300 Zywiec, Poland; 3 Department of Histology, Cytophysiology and Embryology, Faculty of Medicine, Academy of Silesia, 41-800 Zabrze, Poland; 4 Woman and Child Medical Center in Cracow, 30-002 Cracow, Poland; 5 Department of Gynaecology and Obstetrics Faculty of Medicine, Academy of Silesia, 41-800 Zabrze, Poland; 6 Medical Center Dormed Medical SPA, 28-105 Busko-Zdroj, Poland; 7 Department of Gynecology and Obstetrics with Gynecologic Oncology, Ludwik Rydygier Memorial Specialized Hospital, 31-826 Cracow, Poland; 8 Department of Gynecology and Obstetrics, Faculty of Medicine and Health Sciences, Andrzej Frycz Modrzewski University in Cracow, 30-705 Cracow, Poland; 9 Department of Gynecology and Obstetrics, TOMMED Specjalisci od Zdrowia, 40-662 Katowice, Poland; 10 Gyncentrum, Laboratory of Molecular Biology and Virology, 40-851 Katowice, Poland

**Keywords:** endometrial cancer, oxidative stress, miRNAs, ELISA, microarrays, expression profile

## Abstract

Reactive oxygen species are formed as by-products of normal cell metabolism. They are needed to maintain cell homeostasis and signaling, which is possible due to defense systems. Disruption of this balance leads to oxidative stress that can induce cancer. Redox regulation by miRNAs may be a potential therapeutic target. The aim of the study was to assess the activity of genes associated with oxidative stress in endometrial cancer and to determine their relationship with miRNAs. The study included 45 patients with endometrioid endometrial cancer and 45 without neoplastic changes. The expression profile of genes associated with oxidative stress was determined with mRNA microarrays, RT-qPCR and ELISA. The miRNA prediction was performed based on the miRNA microarray experiment and the mirDB tool. PRDX2 and AQP1 showed overexpression that was probably not related to miRNA activity. A high level of PKD2 may be the result of a decrease in the activity of miR-195-3p, miR-20a, miR-134. A SOD3 level reduction can be caused by miR-328, miR-363. In addition, miR-363 can also regulate KLF2 expression. In the course of endometrial cancer, the phenomenon of oxidative stress is observed, the regulation of which may be influenced by miRNAs.

## 1. Introduction

Reactive oxygen species (ROS) are mainly produced by the mitochondria under both physiological and pathological conditions. Their production is based on both enzymatic and non-enzymatic reactions [1,2]. Endogenous production may be associated with inflammation, infection, activation of immune cells, aging, and even cancer. Exogenous production of free radicals may, in turn, be a by-product resulting from exposure to heavy metals, drugs, alcohol or radiation [3,4]. Depending on the level, free radicals can have a beneficial effect on the body. They are involved in cell signaling [5] as well as in the elimination of intracellular pathogens and cancer cells [6]. Interestingly, phagocytes synthesize and store free radicals to destroy invading pathogenic microbes [7]. The proper course of apoptosis, differentiation, activation of transcription factors or protein phosphorylation depends on the low level of ROS in cells [8]. The increase in their production negatively affects proteins, lipids and nucleic acids [9].

Oxidative stress is therefore caused by an imbalance between the production and accumulation ROS in cells and the ability of the biological system to detoxify them. If left unchecked, it can accelerate aging and induce neurodegenerative and cardiovascular diseases, and even cancer [10]. Oxidation of DNA results in the formation of hydrolyzed DNA bases, which impairs cell growth by altering the gene expression profile and promoting the occurrence of gene mutations. In addition, damage to the DNA structure may occur, which promotes the formation of cancer [11,12]. It is important that antioxidant systems, such as molecules that scavenge ROS, heme and iron-dependent proteins or drug-metabolizing enzymes, function properly [13], as it enables cells to efficiently adapt to oxidative stress [14].

ROS may therefore contribute to tumor induction and survival, as well as to treatment resistance [15], but their consistently high levels have a cytotoxic effect, which may be helpful in anticancer therapy [16]. The potential relationship of ROS with microRNAs (miRNAs) is also interesting. These non-coding RNA molecules post-transcriptionally modulate gene expression and can act as oncogenes or tumor suppressors, affecting cancer development, metastasis or survival [17]. The possibility of redox regulation by miRNAs may be a potential therapeutic target in breast cancer [18], hepatocellular carcinoma [19], lung cancer [20], renal cell carcinoma [21], prostate cancer [22].

Endometrial cancer (EC) is one of the most frequently diagnosed gynecological malignancies in the world. It occurs mainly in postmenopausal women, although the number of younger women affected increases every year [23]. Almost half of EC cases are related to obesity. Lifestyle changes, including exercise and a healthy diet, are therefore recommended to lower the risk of this cancer [24]. The diagnosis of endometrial cancer is based on classification systems that take into account both genetic and histological features [25]. However, the complexity of carcinogenesis requires further research to improve the current diagnosis of endometrial cancer and find new therapeutic targets.

The aim of the study was to assess the activity of genes associated with oxidative stress in endometrial cancer and to determine their relationship with miRNAs.

## 2. Results

### 2.1. Oxidative Stress-Related Gene Expression Profile Determined by mRNA Microarrays

A one-way ANOVA with the following Tukey’s post hoc test revealed that out of 600 mRNAs representing oxidative stress-related genes, the number of mRNAs differentiating each cancer grade from the control was as follows: G1 vs. C, 56 mRNAs; G2 vs. C, 112 mRNAs; G3 vs. C, 118 mRNA (*p* < 0.05; FC > 2 or FC < −2). Further analysis indicated that 17 mRNAs were characteristic of G1 cancer, 48 mRNAs for G2 cancer and 56 mRNAs for G3 cancer. In addition, the expression of 25 mRNAs significantly changed regardless of endometrial cancer grade.

The next step involved the overrepresentation test for these 25 common mRNAs representing 18 genes and the selection of the “cellular response to reactive oxygen species” biological process and its subprocesses (Table 1).

A total of 4 out of 18 genes were not classified as related to oxidative stress. On the other hand, *PRDX2*, *PKD2*, *AQP1*, *SOD3*, *KLF2* participate in all subprocesses related to the response to oxidative stress. Detailed results of the microarray experiment for 14 genes associated with oxidative stress are presented in Table 2.

The experiment showed that *AQP1*, *CYBA*, *MELK*, *PKD2*, *PRDX2* were significantly overexpressed in endometrial cancer, while *ATP2B4*, *FOXO1*, *KCNMA1*, *KLF2*, *PRNP*, *SNCA*, *SOD3*, *THBS1*, and *TXNIP* were downregulated.

### 2.2. PRDX2, PKD2, AQP1, SOD3, KLF2 Expression Profile Determined by RT-qPCR and ELISA

The results of the microarray experiment were first verified by RT-qPCR. The Shapiro–Wilk test confirmed the lack of normal distribution of the data. The Kruskal–Wallis and Dunn’s tests were performed. Table 3 shows the results of the statistical analysis together with the median, first (Q1) and third (Q3) quartiles.

The results showed the overexpression of *PRDX2*,* PKD2*, and *AQP1* with reduced levels of *SOD3* and *KLF2*, which is consistent with the microarray experiment.

Then, the expression of *PRDX2*, *PKD2*, *AQP1*, *SOD3*, and *KLF2* was also determined at the protein level (Table 4).

The obtained results indicate the overexpression of PRDX2 and PKD2 in endometrial cancer, regardless of its grade, which is consistent with the analysis at the mRNA level. Similar results were found for KLF2, whose expression was downregulated. In the case of SOD3 and AQP1, their expression was significantly altered in G2 and G3 cancer.

### 2.3. miRNA Target Prediction

Of the 1105 miRNAs found on the microarray, the number of miRNAs differentiating each cancer grade from the control was as follows: G1 vs. C, 131 miRNAs; G2 vs. C, 58 miRNAs; G3 vs. C, 84 miRNAs (*p* < 0.05; FC > 2 or FC < −2). The next step was to assess which of the differentiating miRNAs could participate in the regulation of the activity of PRDX2, PKD2, AQP1, SOD3, and KLF2 (Table 5).

The obtained results indicate that overexpression of *PKD2* may be related to significantly reduced activity of miR-195-3p, miR-20a and increased the levels of miR-106a, miR-328 in the early stages of endometrial cancer. At a later stage, the involvement of miR-134 is also possible. Interestingly, miR-183 initially shows a decrease in activity, which changes dramatically in G3 cancer. The reduced expression of *SOD3* may be due to the increased activity of miR-328 in G1 cancer and miR-363 in G3 cancer. In the case of *KLF2*, miR-195-3p level was reduced while miR-363 was overexpressed. *PRDX2* and *AQP1* expression is most likely not regulated by miRNAs selected in microarray analysis with our criteria.

## 3. Discussion

ROS are formed as by-products of normal cell metabolism. They are needed to maintain cell homeostasis and signaling, which is possible due to defense systems that keep ROS at a low level by converting free radicals into stable, less harmful molecules. Disruption of this balance leads to a phenomenon called oxidative stress [26].

In our study, we analyzed the expression profile of genes related to oxidative stress in endometrial cancer at both mRNA and protein levels. The use of the GeneCards database and the following Overrepresentation test using the Protein Analysis Through Evolutionary Relationship (PANTHER) tool allowed the selection of five genes that were present in all subprocesses related to the cellular response to ROS. An important step was also the identification of miRNAs significantly differentiating endometrial cancer from the control and further prediction of which of them may regulate their expression of peroxiredoxin 2 (PRDX2), polycystin 2 (PKD2), aquaporin 1 (AQP1), superoxide dismutase 3 (SOD3), and Krueppel-like factor 2 (KLF2).

The generation of ROS in cancer cells has been associated with the promotion of proliferation through increased activity of PI3K/Akt signaling as a result of PTEN inactivation [27]. The Akt pathway also promotes cell survival by inactivating the pro-apoptotic transcription factors Bad, Bax, Bim and Foxo [28]. Interestingly, elevated intracellular Ca^2+^ is also associated with the activation of pro-survival pathways [29]. Oxidative stress responses therefore require an influx of Ca^2+^ from both extracellular and intracellular environments as the initial step in combating cellular damage [30]. PKD1 is a large transmembrane sensor protein that interacts with PKD2, a Ca^2+^ permeable cation channel [31].

In our work, PKD2 was significantly overexpressed regardless of cancer grade, which could be due to the miRNA activity. In the early stages of the disease, miR-106a, miR-195-3p and miR-20a appear to be important. Lu et al. observed that miR-106a promotes prostate cancer proliferation through PTEN [32]. Similar conclusions were reached by Xie et al. in the non-small cell lung cancer (NSCLC) study [33]. In turn, Li et al. indicated the promotion of pancreatic cancer metastasis by targeting TIMP-2 [34]. Interestingly, miR-106a acts as a tumor suppressor in renal target carcinoma [35] and colorectal cancer [36]. For endometrial cancer, Tang et al. observed elevated miR-106a level in cell lines (HEC-1-A, HEC-1B, RL95-2, AN3CA, Ishikawa, and JEC), which promotes tumor growth by targeting BCL2L11 [37]. Similar conclusions were drawn by Li et al. in a study on the EC cell line (RL95-2), where overexpression of this miRNA promoted proliferation and inhibited apoptosis [38]. For endometrial cancer tissue samples, studies to date have also shown overexpression of miR-106a [39,40,41], which is consistent with our observations. High levels of miR-106a in G1 endometrial cancer and overexpression of PKD2 may indicate that the potential regulation of expression does not occur directly, and perhaps the activity of elements modulating the normal level of PKD2 is suppressed. It is also possible that there is a loss of the regulatory effects of miR-195-3p and miR-20a, which are silenced in the early stages of the disease. For both miRNAs, our results are consistent with previous studies in endometrial cancer [42,43,44]. High levels of PKD2 in endometrial cancer may also be associated with a significant decrease in miR-134 activity in G2 and G3 cancer. This is not consistent with the study by Hiroki et al. However, it should be noted that our study material was endometrioid EC and not serous EC [42]. MiR-134 is considered a tumor suppressor in gastric cancer [45], esophageal squamous cell carcinoma [46] as well as colorectal cancer [47]. An interesting case is miR-183, whose activity is initially reduced and then dramatically increases in G3 cancer. Furthermore, studies by other researchers indicate that in endometrial cancer, it may be an oncogene as well as a tumor suppressor [48].

In our study, we also noted a reduced expression of SOD3, which may be a consequence of the increased activity of miR-328 and miR-363. SOD3 regulates tissue redox balance and plays a protective role by preventing oxidative damage to proteins and lipids. Low levels of SOD3 are associated with a higher incidence of cancer and poor prognosis [49]. Wang and Xia showed that restoring miR-328 expression in cervical cancer has therapeutic potential [50]. Similarly, in non-small cell lung cancer, the upregulation of this miRNA sensitizes cancer cells to radiotherapy [51]. On the other hand, Wang et al. observed that miR-328 targets PTEN, which confers resistance to cisplatin in NSCLC cells [52]. In addition, targeting LOXL4 promotes growth, survival and migration of this tumor [53]. In the case of miR-363, Lu et al. noted its overexpression in endometrial cancer, which is consistent with our results [54]. In turn, miR-363 also acts as a tumor suppressor in colorectal cancer [55] and gastric cancer [56].

KLF2 regulates the expression of many antithrombotic, antioxidant and anti-inflammatory genes in endothelial cells [57]. Its reduced expression has been reported in colorectal cancer [58], non-small cell lung cancer [59] and prostate cancer [60]. Predictive analysis in our study showed that reduced KLF2 expression could potentially be associated with low miR-195-3p activity and high miR-363 levels, which were described previously.

As part of our research, we also determined the expression profile of PRDX2 and AQP1 at the mRNA and protein levels. Peroxiredoxins are peroxidases that affect differentiation, proliferation and apoptosis [61]. Interestingly, their low levels can sensitize cells to apoptosis and chemotherapy, while their excess promotes resistance to radiation and chemotherapeutic agents [62]. Feng et al. observed that overexpression of PRDX2 inhibited the migration of colorectal cancer cells, which is the result of blocking the epithelial-mesenchymal transition (EMT) [63]. On the other hand, Zheng et al. showed that PRDX2 promotes the progression of this tumor by activating the p38 MAPK pathway [64]. In turn, Chen et al. reported that a high level of PRDX2 promotes a poor prognosis in patients with lung cancer [65]. Aquaporin 1 is a small hydrophobic transmembrane protein that plays a dominant role in transcellular water transport. It is also believed to have a significant role in the migration and invasion of cancer cells [66]. Ji et al. demonstrated that the knockdown of AQP1 inhibited growth, migration and invasion of triple-negative breast cancer [67]. Yamazato et al. showed that AQP1 affects death receptor signaling, resulting in the inhibition of apoptosis and poor prognosis [68]. In turn, Huo et al. observed that hypoxia upregulates AQP1 expression, which contributes to the change of the tumor phenotype to migratory [69].

In the case of PRDX2 and AQP1, further analysis failed to identify miRNAs potentially interacting with these genes. It is possible that they did not meet our criteria or they were not included in our version of the microarrays. It would be promising to expand the list of analyzed miRNAs, as well as to experimentally verify the found interactions. Nevertheless, as part of this work, genes associated with oxidative stress were selected for which a comprehensive analysis was performed, and potential targets for further research were outlined.

## 4. Materials and Methods

The following study was approved by the Bioethical Committee operating at the Regional Medical Chamber in Krakow, no. 185/KBL/OIL/2020 and 186/KBL/OIL/2020, 20 September 2020. All procedures involving human participants were performed in accordance with the guidelines of the 2013 Declaration of Helsinki. Informed consent was obtained from every patient.

All patients enrolled in the study were qualified for hysterectomy. The control group consisted of 45 patients without cancer who underwent surgery for uterine prolapse. A total of 45 patients diagnosed with endometrioid endometrial cancer (EEC) constituted the study group. The collected cancer tissue samples were divided into three subgroups (grades) based on histopathological evaluation: G1, 15 samples; G2, 15 samples; G3, 15 samples. The exclusion criteria included the diagnosis of cancer different than EEC, the coexistence of another cancer, endometriosis, the use of hormone therapy 24 months prior to surgery.

The obtained samples were stored in tubes with Allprotect Tissue Reagent (Qiagen GmbH, Hilden, Germany). TRIzol reagent (Invitrogen Life Technologies, Carlsbad, CA, USA) was used to extract total RNA.

The expression profile of genes involved in oxidative stress was assessed using HG-U133A 2_0 oligonucleotide microarrays (Affymetrix, Santa Clara, CA, USA) and the GeneChip™ HT 3′IVT PLUS kit (Thermo Fisher Scientific, Inc., Waltham, MA, USA) according to the manufacturer’s instruction. The list of genes was prepared based on data from the GeneCards database (https://www.genecards.org/), using the keywords: oxidative AND stress, accessed on 25 August 2022 [70,71]. Then, for genes differentiating endometrial cancer from control, regardless of its grade, the Binomial Overrepresentation test with Bonferroni correction was performed using the Protein Analysis Through Evolutionary Relationship (PANTHER) tool. From GO Biological Process, “cellular response to reactive oxygen species” was selected [72].

RT-qPCR was then performed to validate the results. The SensiFast SYBR No-ROX One-Step Kit (Bioline, London, UK) and β-actin (ACTB), an endogenous control, were used to evaluate the expression profile of *PRDX2*, *PKD2*, *AQP1*, *SOD3*, *KLF2* using a DNA Engine Opticon detector system (MJ Research Inc., Watertown, MA, USA). The thermal profile included reverse transcription (45 °C, 10 min), polymerase activation (95 °C, 2 min) and 40 cycles of denaturation (95 °C, 5 s), hybridization (60 °C, 10 s), and elongation (72 °C, 5 s). Primer sequences are listed in Table 6.

The expression profile of PRDX2, PKD2, AQP1, SOD3, and KLF2 proteins was assessed by ELISA according to the manufacturer’s instructions (Abbexa, Cambridge, UK) using the following kits: Human Peroxiredoxin 2 (PRDX2) ELISA Kit, Human Polycystin-2 (PKD2) ELISA Kit, Human Aquaporin 1 (AQP1) ELISA Kit, Human Superoxide Dismutase 3, Extracellular (SOD3) ELISA Kit, Human Krueppel-like factor 2 (KLF2) ELISA Kit. An Infinite M200 PRO microplate reader (Tecan, Männedorf, Switzerland) was used to evaluate absorbance at 540 nm.

The next step was to determine miRNAs significantly changing expression in endometrial cancer. For this purpose, miRNA 2.0 microarrays (Affymetrix, Santa Clara, CA, USA), FlashTag Biotin HSR RNA Labeling Kit (Affymetrix, Santa Clara, CA, USA) and Hybridization Wash and Stain Kit (Affymetrix, Santa Clara, CA, USA) were used in accordance with the manufacturer’s instructions. The mirDB tool (http://mirdb.org; accessed on 16 October 2022) was then used to predict miRNA targets among the studied genes (target score ≥ 75) [73].

Transcriptome Analysis Console software (Thermo Fisher Scientific, Waltham, MA, USA) was used to analyze the results of the microarray experiments. One-way ANOVA and Tukey’s post hoc test were performed (*p* < 0.05; FC > 2 or FC < −2). Analysis of RT-qPCR and ELISA results was conducted on R using RStudio (version 116 4.2.0, RStudio, Inc., Boston, MA, USA). Data distribution was examined with the Shapiro-Wilk test. The lack of distribution normality allowed the Kruskal–Wallis and Dunn’s post-hoc tests to be performed. A *P*-value adjustment for multiple testing was carried out with the Benjamini–Hochberg false discovery rate correction.

## Figures and Tables

**Table 1:** List of genes involved in response to oxidative stress.

Biological Process	Number of Genes	Fold Change	Gene Symbol	*p*-Value
cellular response to reactive oxygen species	5	58.66	*PRDX2*, *PKD2*, *AQP1*, *SOD3*, *KLF2*	0.0002
response to reactive oxygen species	6	49.31	*PRDX2*, *PKD2*, *AQP1*, *SOD3*, *KLF2*, *TXNIP*	<0.0001
response to oxygen-containing compound	13	11.50	*PRDX2*, *PKD2*, *AQP1*, *SOD3*, *KLF2*, *TXNIP*, *KCNMA1*, *ATP2B4*, *CYBA*, *SNCA*, *THBS1*, *FOXO1*, *PRNP*	<0.0001
response to oxidative stress	10	37.40	*PRDX2*, *PKD2*, *AQP1*, *SOD3*, *KLF2*, *TXNIP*, *SNCA*, *FOXO1*, *PRNP*, *MELK*	<0.0001
cellular response to oxidative stress	8	49.46	*PRDX2*, *PKD2*, *AQP1*, *SOD3*, *KLF2*, *SNCA*, *FOXO1*, *MELK*	<0.0001
cellular response to chemical stress	8	40.22	*PRDX2*, *PKD2*, *AQP1*, *SOD3*, *KLF2*, *SNCA*, *FOXO1*, *MELK*	<0.0001
cellular response to oxygen-containing compound	10	12.96	*PRDX2*, *PKD2*, *AQP1*, *SOD3*, *KLF2*, *ATP2B4*, *CYBA*, *SNCA*, *FOXO1*, *PRNP*	<0.0001

**Table 2:** List of mRNAs representing genes significantly differentiating endometrial cancer from control regardless of its grade, involved in response to oxidative stress (*p* < 0.05).

ID	mRNA	Fold Change		
		G1 vs. C	G2 vs. C	G3 vs. C
207542_s_at	AQP1	3.77	5.05	3.54
209047_at	AQP1	2.9	4.39	2.01
212135_s_at	ATP2B4	−2.77	−3.55	−4.59
212136_at	ATP2B4	−3.43	−3.66	−3.48
203028_s_at	CYBA	2.23	3.26	2.98
202723_s_at	FOXO1	−5.1	−8.03	−7.99
202724_s_at	FOXO1	−3.27	−5.4	−3.89
221583_s_at	KCNMA1	−6.41	−6.43	−5.65
221584_s_at	KCNMA1	−17.35	−18.23	−11.35
219371_s_at	KLF2	−2.66	−5.13	−3.25
204825_at	MELK	3.17	3.84	3.07
203688_at	PKD2	2.26	3.61	3.75
39729_at	PRDX2	2.61	3.18	2.45
201300_s_at	PRNP	−2.89	−8.14	−4.09
215707_s_at	PRNP	−2.2	−3.75	−4.25
204466_s_at	SNCA	−2.42	−4.62	−4.29
205236_x_at	SOD3	−2.47	−2.15	−2.49
201108_s_at	THBS1	−2.4	−3.57	−5.01
201009_s_at	TXNIP	−2.16	−3.58	−4.27

ID—number of the probe; C—control; G—grade of endometrial cancer.

**Table 3:** Values of descriptive statistics, Kruskal–Wallis and Dunn’s tests in endometrial cancer and control (*p* < 0.05).

Gene	Group	mRNA Copies/μg Total RNA			Kruskal–Wallis Test	Dunn’s Test
		Me	Q1	Q3		
*PRDX2*	C	22,980	20,343	25,632	<0.001	G1 vs. C, *p* < 0.001 G2 vs. C, *p* < 0.001 G3 vs. C, *p* < 0.001 G2 vs. G1, *p* = 0.014
	G1	41,321	39,469	44,499		
	G2	51,326	49,250	56,410		
	G3	46,452	39,820	48,895		
*PKD2*	C	19,340	15,375	22,048	<0.001	G1 vs. C, *p* = 0.001 G2 vs. C, *p* < 0.001 G3 vs. C, *p* < 0.001 G3 vs. G1, *p* = 0.003
	G1	32,501	29,793	38,531		
	G2	43,033	38,637	48,262		
	G3	49,906	46,579	58,913		
*AQP1*	C	7804	5050	10,637	<0.001	G1 vs. C, *p* = 0.001 G2 vs. C, *p* < 0.001 G3 vs. C, *p* < 0.001
	G1	12,230	9278	16,038		
	G2	14,683	10,713	17,373		
	G3	15,923	10,938	18,431		
*SOD3*	C	48,162	41,268	51,671	<0.001	G2 vs. C, *p* < 0.001 G3 vs. C, *p* < 0.001 G2 vs. C, *p* < 0.001
	G1	15,368	12,215	17,756		
	G2	11,203	9825	14,341		
	G3	11,823	8284	18,383		
*KLF2*	C	82,257	77,981	87,367	<0.001	G1 vs. C, *p* = 0.001 G2 vs. C, *p* < 0.001 G3 vs. C, *p* < 0.001 G3 vs. G1, *p* = 0.005
	G1	19,671	18,163	23,383		
	G2	13,900	9285	17,330		
	G3	8900	3908	10,063		

Me—median; Q1—lower quartile; Q3—upper quartile; C—control; G—grade of endometrial cancer.

**Table 4:** Concentration of PRDX2, PKD2, AQP1, SOD3, and KLF2 in the study and control groups (*p* < 0.05).

Concentration of Protein [ng/mL]	C	G1	G2	G3
PRDX2	0.45 ± 0.27	0.92 ± 0.12 *	1.01 ± 0.08 *	0.97 ± 0.08 *
PKD2	6.63 ± 1	9.06 ± 1.32 *	10.7 ± 1.32 *	11.1 ± 1.44 *^,#^
AQP1	5.23 ± 0.73	6.90 ± 1.42	8.21 ± 0.90 *	8.20 ± 1.03 *
SOD3	138.85 ± 14.8	69.2 ± 9.66	45.1 ± 6.19 *	32.0 ± 9.46 *^,#^
KLF2	5.79 ± 0.68	2.83 ± 0.36 *	2.52 ± 0.50 *	2.16 ± 0.64 *

C—control; G—grade of endometrial cancer. * *p* < 0.05 vs. C group; # *p* < 0.05 vs. G1 group.

**Table 5:** List of selected genes associated with oxidative stress whose activity may be regulated by miRNAs in endometrial cancer.

mRNA	Expression	miRNA	Fold Change		
			G1 vs. C	G2 vs. C	G3 vs. C
PKD2	upregulated	miR-106a	9.33 *	1.1	1.91
		miR-195-3p	−2.1 *	−1.52	−1.67
		miR-20a	−25.77 *	−1.01	1.06
		miR-134	−3.15	−6.02 *	−16.07 *
		miR-183	−1.49	−6.02 *	79.9 *
SOD3	downregulated	miR-328	7.74 *	1.87	1.24
		miR-363	−1.21	2.14	34.47 *
KLF2	downregulated	miR-195-3p	−2.1 *	−1.52	−1.67
		miR-363	−1.21	2.14	34.47 *

C—control; G—grade of endometrial cancer. * *p* < 0.05 vs. C group.

**Table 6:** RT-qPCR primers.

mRNA	RT-qPCR Amplification Primers (5′-3′)
*PRDX2*	Forward: CCTTCCAGTACACAGACGAGCA Reverse: CTCACTATCCGTTAGCCAGCCT
*PKD2*	Forward: AAATGTCTGGCTGGACCGAGGA Reverse: GGAATCACACCACCTGTTGCTG
*AQP1*	Forward: TATGCGTGCTGGCTACTACCGA Reverse: GGTTAATCCCACAGCCAGTGTAG
*SOD3*	Forward: ACGCTGGCGAGGACGACCTG Reverse: GCTTCTTGCGCTCTGAGTGCTC
*ACTB*	Forward: TCACCCACACTGTGCCCATCTACGA Reverse: CAGCGGAACCGCTCATTGCCAATGG

PRDX2—peroxiredoxin 2; PKD2—polycystin-2; AQP1—aquaporin 1; SOD3—superoxide dismutase 3; KLF2—Krueppel-like factor 2; ACTB—β-actine.

## Data Availability

The data used to support the findings of this study is included in the article. The data will not be shared due to third-party rights and commercial confidentiality.
